# Bicyclol Attenuates Liver Inflammation Induced by Infection of Hepatitis C Virus *via* Repressing ROS-Mediated Activation of MAPK/NF-κB Signaling Pathway

**DOI:** 10.3389/fphar.2018.01438

**Published:** 2018-12-19

**Authors:** Hu Li, Jian-Rui Li, Meng-Hao Huang, Jin-Hua Chen, Xiao-Qin Lv, Li-Li Zou, Jia-Li Tan, Biao Dong, Zong-Gen Peng, Jian-Dong Jiang

**Affiliations:** ^1^ Institute of Medicinal Biotechnology, Chinese Academy of Medical Sciences & Peking Union Medical College, Beijing, China; ^2^ Key Laboratory of Biotechnology of Antibiotics, The National Health and Family Planning Commission (NHFPC), Institute of Medicinal Biotechnology, Chinese Academy of Medical Sciences & Peking Union Medical College, Beijing, China; ^3^ State Key Laboratory of Bioactive Substance and Function of Natural Medicines, Institute of Materia Medica, Chinese Academy of Medical Sciences & Peking Union Medical College, Beijing, China

**Keywords:** bicyclol, hepatitis C virus, anti-inflammatory therapy, inflammatory factor, oxidative stress

## Abstract

Treatment with direct-acting antivirals (DAAs) cures most patients infected with hepatitis C virus (HCV) in the real world. However, some patients, especially those with the underlying advanced liver disease, have a limited reduction of liver injury after achieving a sustained viral response (SVR). Bicyclol was widely used in clinics for the treatment of a variety of liver injuries but with an unknown mechanism for the treatment of hepatitis C. We investigated the anti-inflammatory effects and mechanisms of bicyclol in HCV-infected hepatocytes and further confirmed the putative results in a mouse hepatitis model induced by the coinjection of polyinosinic-polycytidylic acid [poly (I:C)] and D-galactosamine (D-GalN). The results showed that the activation of nuclear factor kappa B (NF-κB) and the subsequent increase of inflammatory factors were directly induced by HCV infection and were persistent after clearance of the virus in Huh7.5 cells. Bicyclol decreased the activation of NF-κB and the levels of inflammatory factors in HCV-infected hepatocytes by inhibiting the activation of the ROS-MAPK-NF-κB pathway, and the effect was synergistic with DAAs in HCV-infected hepatocytes. Bicyclol attenuated the ROS-MAPK-NF-κB axis *via* recovering mitochondrial function without a dependence on dihydronicotinamide adenine dinucleotide phosphate oxidase and superoxide dismutases. The anti-inflammatory effects and mechanism of bicyclol were verified in mouse hepatitis induced by the coinjection of poly(I:C)/D-GalN. Bicyclol directly ameliorates the chronic inflammation caused by HCV infection and might be used with DAAs or after DAA therapy for ultimately curing chronic hepatitis C.

## Introduction

Hepatitis C, caused by infection of hepatitis C virus (HCV), is a worldwide prevailing disease. To date, direct-acting antivirals (DAAs) have substantially improved the sustained viral response (SVR) to above 90% (Jacobson et al., 2017). However, antiviral therapy does not drastically eliminate the liver disease progression, as evidenced by persistent progressive cirrhosis, liver failure, and HCC in a subset of patients who achieved an SVR (Conti et al., 2016; Reig et al., 2016; Jacobson et al., 2017; D’Ambrosio et al., 2018). A most recent cohort of prospective study followed up for 10 years even showed that post-SVR cirrhosis regression does not prevent HCC occurrence (D’Ambrosio et al., 2018). Actually, hepatitis C is associated with inflammation, oxidative stress, and metabolic disorders triggered by HCV infection, which creates the pro-oncogenic microenvironment and results in fibrogenesis, cirrhosis, and HCC (Koike and Miyoshi, 2006; Morgan et al., 2010; Lin et al., 2015; D’Ambrosio et al., 2018). Though early appropriate inflammatory responses are partially beneficial for protecting against pathogens, persistent and uncontrolled inflammatory responses have long-term and repeated malignant follow-up effects in chronic hepatitis C (Nakamoto and Kaneko, 2003; Koike and Miyoshi, 2006; Castello et al., 2010). Therefore, beyond merely achieving an SVR, the prevention of subsequent excessive inflammation, concurrently with other therapeutic regimens, is also imperative for ultimately eliminating hepatitis C (Koike and Miyoshi, 2006; Castello et al., 2010).

Bicyclol (4,4′-dimethoxy-5,6,5′,6′-bis (methylenedioxy)-2-hydroxymethyl-2′-methoxycarbonyl biphenyl) is an approved drug in China, which is used as a hepatoprotective and anti-inflammatory agent (Liu, 2009). Accumulating clinical evidence shows its substantial effects in various liver injuries, including viral hepatitis (Yao et al., 2002; Liu, 2009), with *in vitro* mechanisms related to multiple possible molecules, such as the Toll-like receptor, inosine 5′-monophosphate dehydrogenase II, heat shock protein 70/27, and heme oxygenase (Bao and Liu, 2009; Zhang et al., 2014,^ 2016^). The anti-HCV activity of long-term use of bicyclol was also reported (Liu, 2009). However, unlike the single pathogen-associated molecular patterns or damage-associated molecular pattern signal, HCV infection-mediated inflammatory cytokine and chemokine storms *in vitro* and *in vivo* are acute and sophisticated, and there is still no research about the role and mechanism of bicyclol in hepatitis C. Here we analyzed the effects and mechanisms of bicyclol in HCV-infected hepatocytes and in mouse hepatitis induced by the coinjection of polyinosinic-polycytidylic acid [poly (I:C)] and D-galactosamine (D-GalN), which mimic the vigorous inflammatory state and activation of intracellular signaling pathways during HCV infection. We revealed that bicyclol attenuates liver inflammation induced by HCV infection *via* repressing the ROS-mediated activation of the MAPK/NF-κB signaling pathway.

## Materials and Methods

### Cells and Virus

The hepatocyte Huh7.5 cells and the plasmid pFL-J6/JFH/JC1, containing a full-length chimeric HCV cDNA, were kindly provided by the Vertex Pharmaceuticals Inc. (Boston, USA). The primary human hepatocytes (PHHs) were from the ScienCell Research Laboratories (San Diego, CA, USA). The HCV virus stock was prepared as previously described (Peng et al., 2011). For preparing the virus-free supernatants, the HCV stock was layered onto a 20% sucrose cushion and was ultracentrifuged at 250,000 g for 4 h at 4°C; the virus-free supernatants and DMEM-resuspended virus pellets under the cushion were collected. For preparing UV-inactivated HCV, the HCV stocks were irradiated by UV light at a dose of 20 mj/cm^2^ for 20 min.

### Agents

Bicyclol was from the Beijing Union Pharmaceutical Company (Beijing, China) with purity over 99%. Anti-HCV positive drugs, such as sofosbuvir, simeprevir, and daclatasvir, were from the MedChemExpress (Princeton, NJ, USA). BAY11-7082 (Beyotime Biotechnology, Jiangsu, China), diphenyliodonium (DPI) (Sigma, St. Louis, MO, USA), *N*-acetylcysteine (Beyotime Biotechnology), SB203580 (CST, Beverly, MA, USA), SP600125 (CST), and U0126 (CST) were used in the experiments.

### Cytotoxicity Assay

Huh7.5 cells were treated with drugs or the solvent control for 12 or 72 h. The cell viability was detected with an MTT assay and was calculated as described before (Peng et al., 2010).

### Western Blot Assay

Western blot was performed as previously described (Peng et al., 2010). Briefly, after SDS-PAGE and transmembrane, the target proteins were accordingly probed with antibodies against β-actin (CST, cat#3700s), CuZn-SOD (CST, cat#2770), Mn-SOD (CST, cat#13141), cytochrome c (CST, cat#11940), Nox1 (Boster Biological Technology, cat#BA3335), Nox4 (Abcam, cat#ab133303), HCV Core (Abcam, cat#ab2740), HCV NS3 (Abcam, cat#ab13830), and phospho-specific or total p38, ERK, JNK, or NF-κB p65 (CST, cat#9910, #9926, and #3033). After an incubation with the corresponding HRP-conjugated secondary antibody, the signal of the target proteins was detected using the ChemiDo XRS gel imager system (Bio-Rad), with an enhanced chemiluminescence (ECL) kit (GE Healthcare Life Sciences, Pittsburgh, PA, USA) and was scanned with the Gelpro32 software. The ratio of the protein of interest to the internal control protein Actin was calculated and normalized as 1.00 for the control group.

### Real-Time Quantitative Reverse Transcript PCR

HCV RNA was quantified with real-time quantitative reverse transcript PCR (qRT-PCR), and the result was calibrated with the internal control gene glyceraldehyde 3-phosphate dehydrogenase (GAPDH) as previously reported (Peng et al., 2010). The mRNA of TNF-α, IL-6, and MIP-1β was amplified with specific primers (Table 1). Briefly, total RNA (1 μg) isolated from the cells or tissues was reverse transcribed into cDNA using the GoScript Reverse Transcription System (Promega) and was quantified with the GoTaq qPCR Master Mix (Promega) according to the manufacturer’s protocol. The relative mRNA amounts were calculated by the comparative Ct method after normalizing against the amount of GAPDH mRNA.

**Table 1:** The primers used in qRT-PCR.

Gene	Sense primer (5′~3′)	Antisense primer (5′~3′)
h-GAPDH	CGGAGTCAACGGATTTGGTCGTAT	AGCCTTCTCCATGGTGGTGAAGAC
h-TNF-α	CAGCCTCTTCTCCTTCCTGAT	GCCAGAGGGCTGATTAGAGA
h-IL-6	CAGGAGCCCAGCTATGAACT	AGCAGGCAACACCAGGAG
h-MIP-1β	CAGCGCTCTCAGCACCAATGG	GATCAGCACAGACTTGCTTGCTTC
mus-GAPDH	CTCTGGAAAGCTGTGGCGTGATG	ATGCCAGTGAGCTTCCCGTTCAG
mus-TNF-α	CCAAAGGGATGAGAAGTTCC	CTCCACTTGGTGGTTTGCTA
mus-IL-6	CCATCCAGTTGCCTTCTTGG	TGCAAGTGCATCATCGTTGT
mus-MIP-1β	TGCTCGTGGCTGCCTTCTGT	TGTGAAGCTGCCGGGAGGTGTA

Note: h, human; mus, mouse.

### Luciferase Reporter Assay

Huh7.5 cells were cotransfected with the plasmid pNF-κB-Luc (Promega), expressing firefly luciferase, and the pRL-SV40 vector (Promega), expressing Renilla luciferase, in a 10:1 mass ratio using Lipofectamine 2000 (Invitrogen, Carlsbad, CA, USA). After 24 h of transfection, the cells were pretreated with different inhibitors for 12 h and were then treated with or without 200 μM of H_2_O_2_ for another 2 h. The luciferase activity of the cell lysate was determined with a dual-luciferase reporter assay kit (Beyotime Biotechnology) according to the manufacturer’s protocol. The relative luciferase activities were calculated by the ratio of the intensity of firefly luminescence to the intensity of the reference Renilla luminescence.

### Detection of Reactive Oxygen Species in the Cells and Liver Tissues

The treated cells were incubated with 5 μM dihydroethidium (DHE) (Beyotime Biotechnology) for 30 min at 37°C. After being washed, the cells were resuspended in PBS. The superoxide level was measured by a flow cytometer (Sanchez-Reus et al., 2005). The ROS assay in the liver tissues was performed as previously described (Wang et al., 2014). After fixing and then dehydrating at 4°C, the tissues were embedded using optimal cutting temperature compound (OCT) and were cut into sections of 5 μm thickness. The sections were washed and then incubated with DHE (50 μM) for 60 min followed by washing with PBS three times and were then mounted with an antifade mountant (Invitrogen). The red fluorescence signal was acquired with a fluorescence microscope, and the ROS accumulation was measured and statistically analyzed in three random vision fields by Image-Pro plus 6.0.

### Animal Experiments

The male C57BL/6 mice (20.0 ± 2.0 g) were from the Beijing HuaFuKang Biological Technology Co. Ltd. The mice were randomly divided into five groups, with six mice in each group and were daily administered intragastrically with bicyclol (75, 150, and 300 mg/kg) dissolved in 0.5% carboxymethyl cellulose sodium (CMC-Na) or solvent control for 7 days. The mice were injected intravenously with polyinosinic-polycytidylic acid sodium (poly(I:C), InvivoGen) dissolved in pyrogen-free saline at a dose of 75 μg/kg and were simultaneously intraperitoneally injected with D-galactosamine (D-GalN, Sigma) at a dose of 500 mg/kg. The control group was injected intravenously and intraperitoneally with pyrogen-free saline. Sixteen hours after the injection, the mice were sacrificed, and the blood and liver tissues were collected. The blood samples were centrifuged, and the serum was collected. The liver tissues were stored at −80°C for total RNA and protein extraction or were embedded in 10% formaldehyde and 4% paraformaldehyde for hematoxylin-eosin (H&E) or DHE staining, respectively. Formalin-fixed and paraffin-embedded liver tissues were also used for the monocytes, macrophages and Kupffer cell marker CD68 immunohistochemical (IHC) staining. The distribution of ROS was detected through comparing the paraformaldehyde fixed liver tissue slice in white light, nucleus stained with DAPI, and ROS stained with DHE. Serum alanine transaminase (ALT) and aspartate transaminase (AST) were detected using ALT and AST assay kits (Nanjing Jiancheng Bioengineering Institute, China), respectively. Liver cytokine (TNF-α, IL-6, and MIP-1β) mRNA and protein levels were quantified with qRT-PCR and ELISA kits (USCN Life Science Inc., Wuhan, China), respectively. Animal experiments were conducted following the National Guidelines for Housing and Care of Laboratory Animals and were performed in accordance with a protocol approved by the Institutional Animal Care and Use Committee.

### Isolation of Cytoplasmic and Mitochondrial Proteins

Mitochondria were isolated using a mitochondria isolation kit (Beyotime Biotechnology). Briefly, the cells were homogenized and centrifuged at 600 g for 10 min at 4°C to remove the nuclei. The supernatants were centrifuged again at 11,000 g for 10 min at 4°C to obtain crude mitochondria pellets. The cytosolic proteins were obtained by centrifuging the supernatants for another 10 min at 12,000 g. The proteins were boiled for western blot assay after determining the concentration using a BCA protein assay kit (Thermo).

### Cellular Mitochondrial Membrane Potential Measurement

The mitochondrial membrane potential was measured by flow cytometry using Rho123 (Sigma) staining, as described previously (Wang et al., 2014). Briefly, the cells were incubated with Rho123 (0.5 μg/mL) at 37°C for 25 min and then were harvested and washed twice with PBS, and a total of 10,000 events per sample was measured by a flow cytometer. The fluorescent signal intensity was analyzed with BD CellQuest Pro software.

### Measurement of Cellular NADPH Oxidase and CuZn/Mn-SOD Activity

The cells were collected and lysed, and the dihydronicotinamide adenine dinucleotide phosphate (NADPH) oxidase activity was measured using an NADPH oxidase activity assay kit (Genmed Scientifics Inc., Shanghai, China), which was detected by an EnSpire Multimode Plate Reader (PerkinElmer, Waltham, MA, USA). The CuZn/Mn-SOD activity was measured by the CuZn/Mn-SOD assay kit (Beyotime Biotechnology). The relative activity was normalized to the protein contents and presented as a percent of the control.

### Statistical Analysis

The data are presented as the mean ± standard deviation of over three independent experiments. Analysis of variance (ANOVA) followed by Student-Newman-Keuls (SNK) *post hoc* tests were performed to compare the different parameters between the groups using SPSS17.0. The value of statistical significance was set at *p* < 0.05.

## Results

### NF-κB-Mediated Inflammatory Response Is Directly Induced by HCV Infection and Is Persistent Even After Clearance of the Virus in Huh7.5 Cells

**Figure 1:**
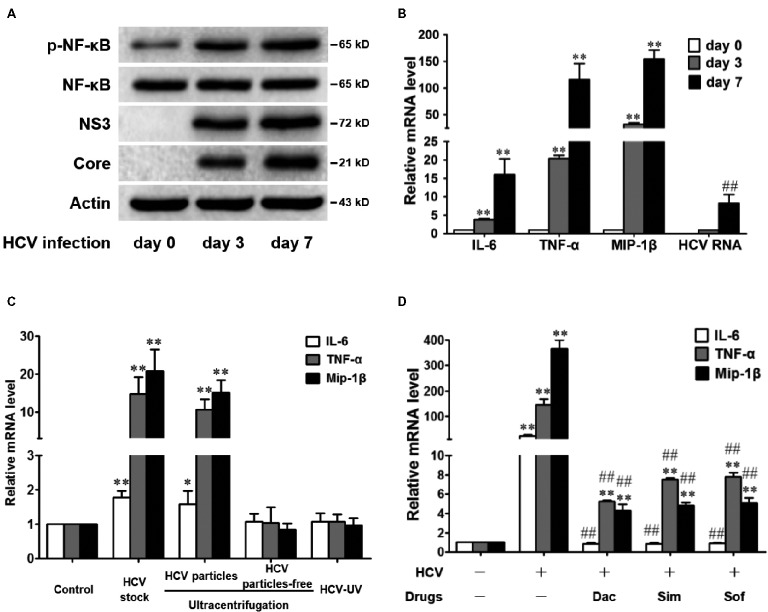
HCV infection induces NF-κB activation and subsequently increases the levels of inflammatory factors in Huh7.5 Cells. **(A,B)** Huh7.5 cells were infected with HCV (MOI = 1). At 0, 3, and 7 days, intracellular protein and RNA were detected by western blot **(A)** and qRT-PCR **(B)**, respectively. **(C)** Huh7.5 cells were infected with infectious HCV (HCV particles obtained by ultracentrifugation or HCV stock, MOI = 2), UV-inactivated HCV (HCV-UV), and virus-free supernatants, and intracellular RNA was detected by qRT-PCR at 48 h. **(D)** Huh7.5 cells, 3 days post infection (MOI = 1), were treated with daclatasvir (Dac, 100 pM), simeprevir (Sim, 0.2 μM), or sofosbuvir (Sof, 0.5 μM) for 8 days, and the intracellular RNA was detected by qRT-PCR. The protein bands show the results of a representative experiment. The experiments were performed at least in triplicate, and each value represents the mean ± SD. ANOVA analysis with the SNK method was used in **(B**–**D)**. *p < 0.05, **p < 0.01 versus the solvent control; ^#^ *P* < 0.05, ^##^ *P* < 0.01 versus the HCV-infected control group.

HCV infection increases the level of intracellular inflammatory cytokines and chemokines, such as tumor necrosis factor-α (TNF-α), interleukin-6 (IL-6), and chemokine macrophage inflammatory protein 1 beta (MIP-1β) *in vivo* and *in vitro* (Zeremski et al., 2007; Lin et al., 2014), and nuclear factor kappa B (NF-κB) activation is a common upstream event that causes the increase. We first verified whether HCV infection directly induced an inflammatory response in an NF-κB-dependent manner *in vitro*. Huh7.5 cells were infected with HCV for 0, 3, and 7 days, and the amount of phosphorylated NF-κB (p-NF-κB) was increased in a time-dependent manner without changing the total NF-κB amount (Figure 1A). Intracellular TNF-α, IL-6, and MIP-1β mRNA levels were also increased (Figure 1B) and were positively related to the HCV viral loads (Figure 1A,B). Therefore, our results verified that HCV infection induced NF-κB activation and the subsequent liver inflammatory response in Huh7.5 cells, which was consistent with previous reports (Lin et al., 2010, 2011, 2014).

To corroborate that the increased inflammatory response was mediated by HCV infection or by the increased inflammatory mediators in the supernatants, we compared the role of infectious HCV (HCV stock or HCV particles obtained by ultracentrifugation), UV-inactivated HCV (HCV-UV), and the virus-free supernatants. The results showed that infectious HCV increased TNF-α, IL-6, and MIP-1β mRNA levels, while the culture supernatants with HCV particle-free or inactivated HCV were unable to show this upregulation (Figure 1C), suggesting that the inflammatory response was exclusively activated by the HCV infection but not the inflammatory mediator in our infectious system.

In the clinic, aggravated liver disease progress after the clearance of HCV is still persistent (Lin et al., 2015; Jacobson et al., 2017). Therefore, we examined the inflammatory state after virus eradication *in vitro*. After the HCV-infected Huh7.5 cells were treated with DAAs for 8 days, HCV RNAs were undetectable (data not shown). However, the TNF-α and MIP-1β mRNA levels were still significantly higher than those in the control cells (Figure 1D), although the IL-6 mRNA decreased to the basal level. The results agree with previous reports in the clinic that anti-HCV therapy does not completely cure HCV-induced inflammatory diseases (Koike and Miyoshi, 2006; Morgan et al., 2010; Lin et al., 2015; D’Ambrosio et al., 2018).

### Bicyclol Decreases the NF-κB Activation and Inflammatory Response in HCV-Infected Hepatocytes

**Figure 2:**
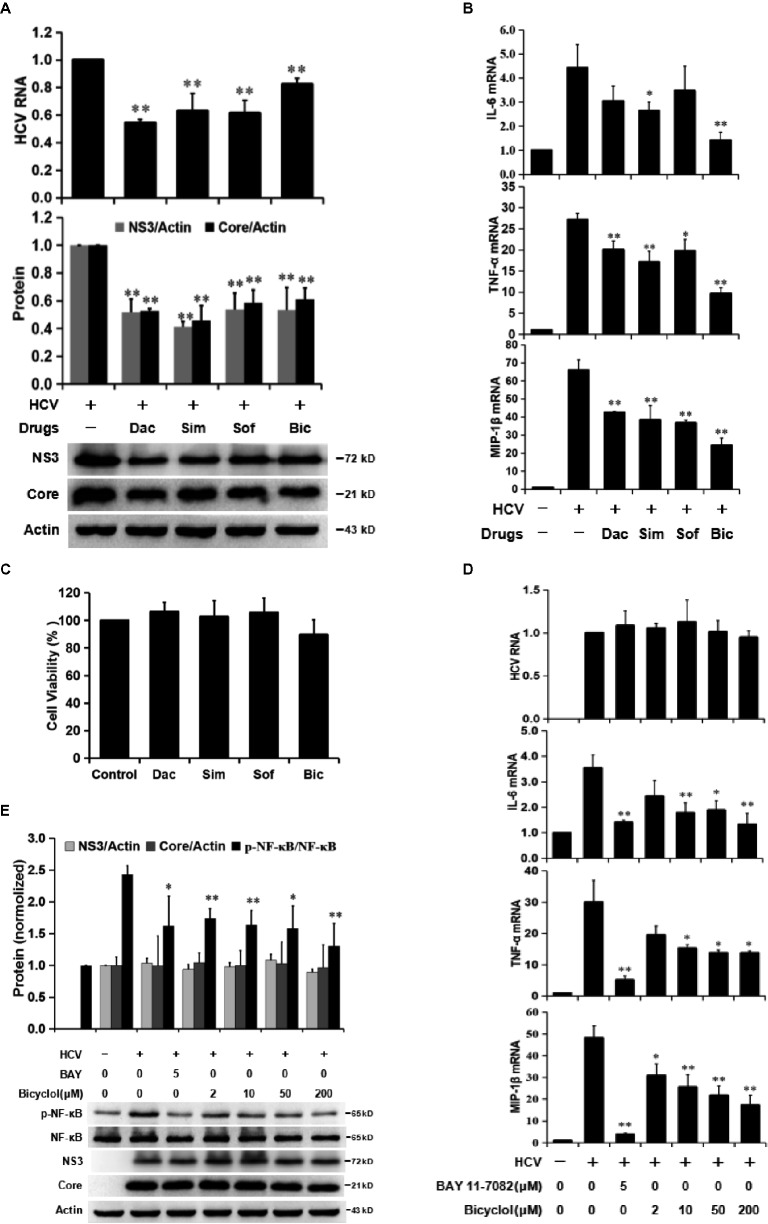

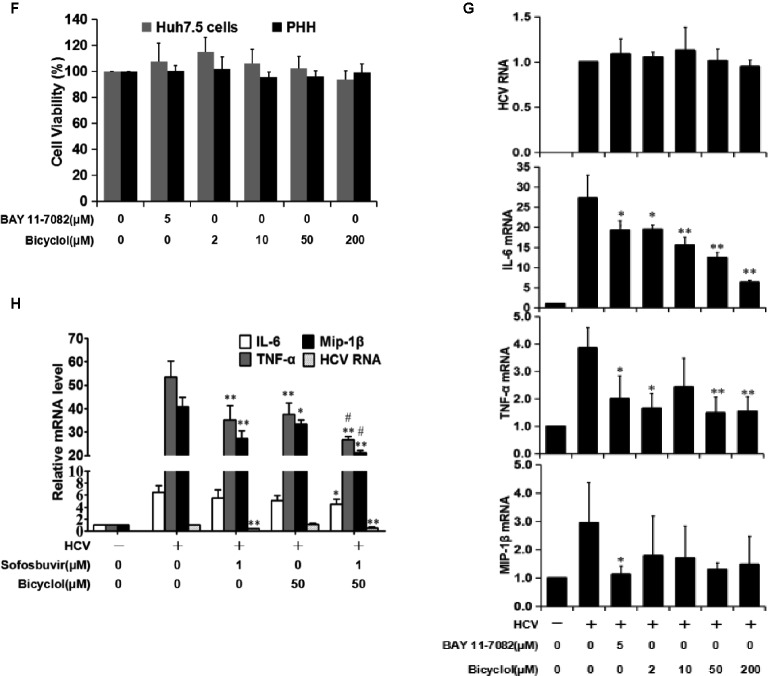
Bicyclol decreases HCV-induced NF-κB activation and the levels of inflammatory factors in hepatocytes. **(A–C)** Huh7.5 cells infected with HCV (MOI = 1) were simultaneously treated with bicyclol (Bic, 100 μM) or DAAs (daclatasvir (Dac, 10 pM), simeprevir (Sim, 0.02 μM), and sofosbuvir (Sof, 0.1 μM)) for 72 h, and intracellular RNA and proteins were detected by qRT-PCR and western blot **(A,B)**, respectively, and the cytotoxicity was detected by MTT assay **(C)**. **(D–F)** HCV-infected Huh7.5 cells were starved for 4 h and were then treated with the NF-κB inhibitor BAY11-7082 or bicyclol for 12 h; the intracellular RNA and proteins were detected by qRT-PCR **(D)** and western blot **(E)**, respectively, and the cytotoxicity was detected by MTT assay **(F)**. **(F–G)** Primary human hepatocytes (PHHs), 4 days post-HCV infection (MOI = 2), were treated with bicyclol for 12 h, and the intracellular RNAs were detected by qRT-PCR **(G)**, and the cytotoxicity was detected by MTT assay **(F)**. **(H)** Huh7.5 cells, 6 days post infection with HCV (MOI = 0.2), were treated with sofosbuvir (1 μM) and/or bicyclol (50 μM) for 24 h, and the intracellular RNA was detected by qRT-PCR. The protein bands show the results of a representative experiment. The experiments were performed at least in triplicate, and each value represents the mean ± SD. ANOVA analysis with the SNK method was used. *p < 0.05, **p < 0.01 versus the HCV-infected control group; ^#^ *p* < 0.05 versus the sofosbuvir-treated group.

Clinical and experimental data show that bicyclol has anti-inflammatory and hepatoprotective effects, and long-term use of bicyclol inhibits HCV replication *via* a new mechanism that is different from that of DAAs (Guo et al., 2003; Liu, 2009). To verify bicyclol’s anti-inflammatory and anti-HCV effects, Huh7.5 cells were infected with HCV and were simultaneously treated with bicyclol or DAAs (daclatasvir, simeprevir, and sofosbuvir) for 72 h. The results showed that both the bicyclol and DAA treatments decreased the levels of HCV RNA and proteins (Figure 2A) and the inflammatory factor TNF-α, IL-6, and MIP-1β mRNA levels (Figure 2B) without cytotoxicity (Figure 2C). However, bicyclol was more effective than the DAAs, with regard to the anti-inflammatory effects, under identical anti-HCV activities (Figure 2A,B).

To further distinguish bicyclol’s anti-inflammatory and anti-HCV effect, we examined whether bicyclol reduces the inflammatory response in HCV-infected Huh7.5 cells within 12 h of treatment. The results showed that TNF-α, IL-6, and MIP-1β mRNA levels were decreased (Figure 2D), and HCV-induced p-NF-κB activation was inhibited by bicyclol (Figure 2E) without changing the HCV RNA and protein levels (Figure 2D,E). Certainly, BAY11-7082, an NF-κB inhibitor, was effective in this model (Figure 2D,E) without cytotoxicity (Figure 2F). The results were similar in HCV-infected primary human hepatocytes (Figure 2G) and were without cytotoxicity (Figure 2F). Furthermore, the combined use of bicyclol with sofosbuvir synergistically reduced the inflammatory factors but not the HCV RNA level in HCV-infected Huh7.5 cells within 24 h of treatment (Figure 2H). These results suggest that bicyclol has a potential anti-inflammatory effect during HCV infection, which is at least partly not due to its anti-HCV effects.

### Bicyclol Attenuates HCV-Induced Activation of NF-κB Through the Inhibition of the ROS-MAPK-NF-κB Pathway in Huh7.5 Cells

**Figure 3:**
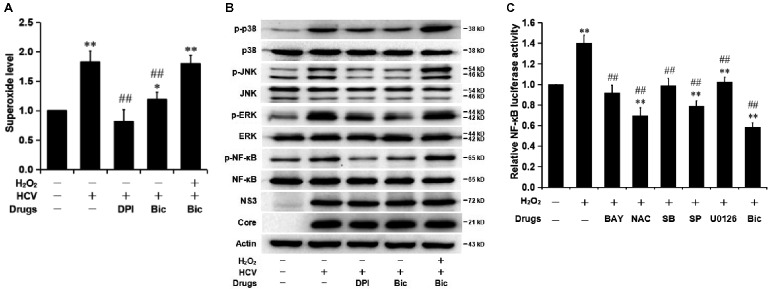
Bicyclol inhibits the HCV-induced activation of NF-κB by inhibiting the activation of the ROS-MAPK-NF-κB pathway in Huh7.5 cells. **(A,B)** Naive and HCV-infected (MOI = 1) Huh7.5 cells were serum starved for 4 h and pretreated with the ROS inhibitor DPI (20 μM) and bicyclol (Bic, 200 μM) for 10 h and were then treated with or without H_2_O_2_ (200 μM) for another 2 h; the intracellular superoxide levels **(A)** and phospho-specific and total p38/ERK/JNK and NF-κB amounts **(B)** were detected. **(C)** Huh7.5 cells were cotransfected with the plasmid pNF-κB-Luc expressing firefly luciferase and the control plasmid, pRL-SV40 vector, expressing Renilla luciferase in a 10:1 mass ratio. After 24 h of transfection, the cells were pretreated with the NF-κB inhibitor BAY11-7082 (BAY, 5 μM), ROS scavenger NAC (10 mM), p38 inhibitor SB203580 (SB, 10 μM), JNK inhibitor SP600125 (SP, 10 μM), ERK inhibitor U0126 (10 μM), or bicyclol (Bic, 200 μM) for 10 h and were then treated with or without H_2_O_2_ (200 μM) for another 2 h. The relative luciferase activity was measured. The experiments were performed at least in triplicate, and each value represents the mean ± SD. ANOVA analysis with the SNK method was used. *p < 0.05, **p < 0.01 versus the solvent control; ^#^ *p* < 0.05, ^##^ *p* < 0.01 versus the HCV- or H_2_O_2_-treated group.

NF-κB activation is commonly related to the activation of its upstream mitogen-activated protein kinases (MAPKs), including p38 MAPK, extracellular signal-regulated kinase (ERK), and Jun N-terminal kinase (JNK), and the upper-stream reactive oxygen species (ROS) (Lin et al., 2010; Hsu et al., 2013). Two types of ROS, superoxide anion (O_2_^•−^) and secondary generated hydrogen peroxide (H_2_O_2_), are induced by HCV protein expression in replicon models and infectious system (Gong et al., 2001; Paracha et al., 2013) and, finally, contribute to the development of liver disease *via* NF-κB activation (Tardif et al., 2005; Lin et al., 2010). Previous reports show that bicyclol has a potent hepatocyte-protective effect partly by decreasing oxidative stress (Liu, 2009), and therefore, we assessed the role of bicyclol in the ROS-MAPK-NF-κB axis during HCV infection. HCV-infected Huh7.5 cells were pretreated with the ROS inhibitor diphenyliodonium (DPI) or bicyclol for 10 h and were then treated with or without H_2_O_2_ for another 2 h. The results showed that bicyclol decreased HCV infection induced cellular O_2_^•-^ levels (Figure 3A) and inhibited the phosphorylation of p38, ERK, JNK, and NF-κB (Figure 3B), with unchanged amounts of HCV protein and total p38, ERK, JNK, and NF-κB (Figure 3B). The effect of bicyclol was similar with that of the ROS inhibitor DPI (Figure 3A,B), which was neutralized by exogenous H_2_O_2_ (Figure 3A,B). These results suggest that the HCV infection-activated ROS-MAPK-NF-κB pathway might be interrupted by bicyclol.

To further investigate the inactive role of bicyclol in the ROS- and MAPK-mediated NF-κB activation, we applied H_2_O_2_ to mimic the induced oxidative stress state by HCV infection. Huh7.5 cells, cotransfected with the plasmid pNF-κB-Luc and control plasmid pRL-SV40, were treated with the NF-κB inhibitor BAY11-7082, ROS scavenger *N*-acetylcysteine (NAC), p38 inhibitor SB203580, JNK inhibitor SP600125, ERK inhibitor U0126, or bicyclol for 10 h and were then stimulated with H_2_O_2_ for another 2 h. Predictably, we observed H_2_O_2_-induced NF-κB activation (Figure 3C), and the NF-κB activity was decreased by all those inhibitors or bicyclol (Figure 3C). These data further verified that bicyclol inhibits the HCV-induced inflammatory response through the ROS-MAPK-NF-κB pathway.

### Bicyclol Decreases the Level of HCV-Induced ROS Through Restoration of Mitochondrial Function Without a Dependence on NADPH Oxidase and Superoxide Dismutases

**Figure 4:**
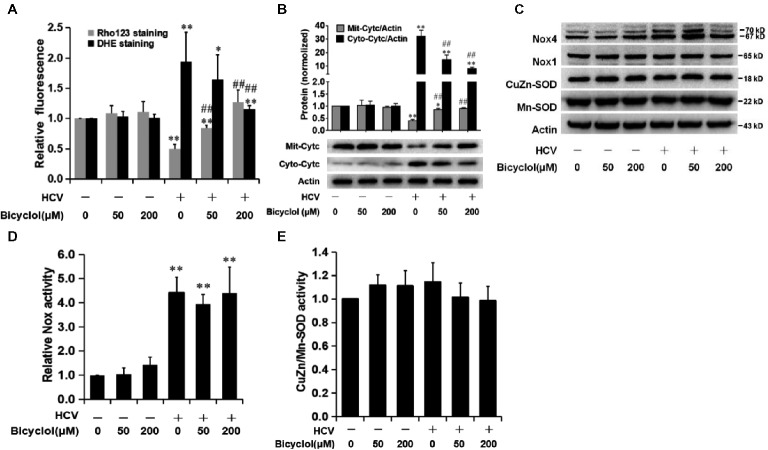
Bicyclol decreases the level of HCV-induced ROS through the restoration of mitochondrial function but is independent of NADPH oxidase and superoxide dismutases. Naive and HCV-infected Huh7.5 cells were treated with bicyclol for 12 h. The cells were stained by Rho123 (0.5 μg/mL) for 25 min to measure the mitochondrial membrane potential or stained by 5 μM dihydroethidium (DHE) for 30 min to measure the superoxide level using a flow cytometer **(A)**. Cytosolic and mitochondrial proteins were extracted, and cytochrome c was detected by western blot **(B)**. Total cellular protein was detected by western blot **(C)**, and the activities of Nox **(D)** and CuZn/Mn-SOD **(E)** were detected with assay kits. The protein bands show the result of a representative experiment. The experiments were performed at least in triplicate, and each value represents the mean ± SD. ANOVA analysis with the SNK method was used. *p < 0.05, **p < 0.01 versus the solvent control; ^#^ *p* < 0.05, ^##^ *p* < 0.01 versus the HCV-infected control group.

Superoxide anion is mainly derived from mitochondrial electron transport chain and cytoplasmic production after dihydronicotinamide adenine dinucleotide phosphate (NADPH) oxidase activation (Brault et al., 2013). Mitochondrial depolarization is widely reported to enhance the generation of O_2_^•-^ (Budd et al., 1997). Therefore, we detected the effect of bicyclol on the mitochondrial transmembrane potential (ΔΨm). HCV-infected Huh7.5 cells were treated with bicyclol for 12 h, and ΔΨm and O_2_^•−^ were detected. The results showed that HCV infection decreased ΔΨm (shown by Rho123 staining) and increased the superoxide anion level (shown by DHE staining) (Figure 4A), which is consistent with a previous report (Deng et al., 2008). Bicyclol reversed these changes in a dose-dependent manner (Figure 4A), suggesting that bicyclol might decrease the level of HCV-induced ROS by restoring HCV-decreased ΔΨm in HCV-infected cells.

The decreased ΔΨm leads to mitochondrial dysfunction, which is characterized by cytochrome c release from mitochondria into cellular cytosol (Deng et al., 2008). Our results showed that mitochondrial cytochrome c was decreased (Figure 4B), while cytosol cytochrome c was increased (Figure 4B) after HCV infection. We also observed a decreased release of cytochrome c after treatment with 50 μM bicyclol for 12 h (Figure 4B), with no effect in naive Huh7.5 cells (Figure 4B), suggesting that the bicyclol decreasing the level of HCV-induced ROS might be through the restoration of mitochondrial function.

NADPH oxidase (Nox) catalyzes the transfer of electrons from NAD(P) H to O_2_ and thus produces O_2_^•−^. The degradation of O_2_^•−^ into H_2_O_2_ relies on mitochondrial or cytosolic superoxide dismutases (Mn-SOD or CuZn-SOD). Previous studies showed that among the seven Nox enzymes (Nox1-5, Duox1, and Duox2), Nox1 and Nox4 proteins might be increased in HCV-infected or HCV core protein expressed cells (Boudreau et al., 2009; de Mochel et al., 2010), while the effect of HCV infection on Mn-SOD or CuZn-SOD was controversial (Abdalla et al., 2005; Lee et al., 2016). Our results showed that HCV only slightly increased the expression of Nox4, but not Nox1 (Figure 4C), while Nox activities were significantly increased after HCV infection (Figure 4D). However, the increased expressions and activities of the Nox were not changed after bicyclol treatment (Figure 4C,D). Meanwhile, the expression and activity of Mn-SOD and CuZn-SOD were not influenced by HCV infection or by bicyclol treatment (Figure 4C,E). These data hint that the decreased level of HCV-induced superoxide anion by bicyclol is independent of NADPH oxidases and superoxide dismutases.

### Bicyclol Reduces Liver Injury and Inflammation *via* Attenuating the Activation of the ROS-MAPK-NF-κB Pathway in Hepatitis Mice

**Figure 5:**
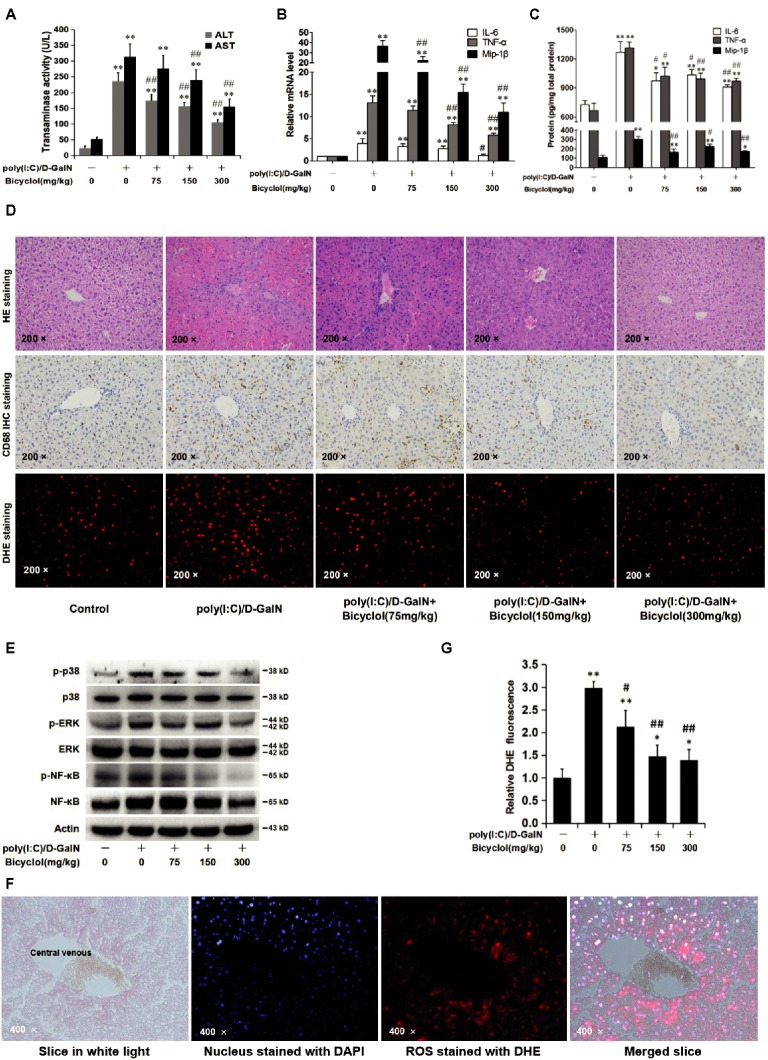
Bicyclol reduces liver injury and inflammation *via* attenuating the activation of the ROS-MAPK-NF-κB pathway in hepatitis mice. Mice (*n* = 6 for each group) were intragastrically administered with bicyclol daily or a solvent control for 7 days, and then, the mice were intravenously injected with poly(I:C) at a dose of 75 μg/kg or a solvent control. Simultaneously, the mice were intraperitoneally injected with D-GalN at a dose of 500 mg/kg or a solvent control. Sixteen hours after the injection, the serum was collected for the ALT and AST assays **(A)**, and the liver tissues were prepared for RNA detection **(B)**, ELISA assay **(C)**, H&E and CD68 IHC staining **(D)**, and western blot **(E)**. The level of ROS in the liver tissues was detected using the fluorescent probe DHE (**D**, DHE staining), and the distribution of ROS was detected through comparing the paraformaldehyde fixed liver tissue slice in white light, nucleus stained with DAPI, and ROS stained with DHE **(F)**. ROS accumulation was measured and analyzed in three random vision fields by Image-Pro plus 6.0 **(F)**. ANOVA analysis with the SNK method was used. *p < 0.05, **p < 0.01 versus the control group; ^#^ *p* < 0.05, ^##^ *p* < 0.01 versus the poly(I:C)/D-Gal treated group.

Simultaneous injection of poly(I:C) and D-GalN induces serious liver damage in mice, and thus, this rodent animal model is used to evaluate the protective role of the candidates (Huang et al., 2016). The levels of ALT and AST in the serum were increased after the coinjection of poly(I:C)/D-GalN (Figure 5A), suggesting that the liver was injured. However, the transaminase level was markedly decreased in a dose-dependent manner by an intragastric administration of bicyclol (Figure 5A). The mRNA (Figure 5B) and protein levels (Figure 5C) of liver tissues TNF-α, IL-6, and MIP-1β were significantly increased in the poly(I:C)/D-GalN group (Figure 5B,C), while bicyclol decreased the inflammation in a dose-dependent manner (Figure 5B,C). Histopathological findings also revealed that poly(I:C)/D-GalN induced spotted necrosis and inflammatory infiltration in the liver, and bicyclol alleviated these pathologic changes (Figure 5D, HE staining and CD68 IHC staining). These data suggest that bicyclol improves poly(I:C)/D-GalN-induced hepatitis in mice, which is consistent with the clinical data (Yao et al., 2002; Liu, 2009).

D-GalN or poly(I:C) triggers ROS production, MAPK pathway activation, and inflammatory response *in vitro* and *in vivo* (Dejager and Libert, 2008; Liu et al., 2008; Lin et al., 2009). We detected changes in the ROS-MAPK-NF-κB axis in the mouse liver. As shown in Figure 5E, poly(I:C)/D-GalN increased phosphorylated-MAPK (p-p38 and p-ERK) levels, while the p-JNK was not detectable, which might be due to the low level of p-JNK in this model. In the bicyclol-treated group, the levels of p-p38, p-ERK, and downstream p-NF-κB were obviously downregulated (Figure 5E). The distribution of liver cells (nucleus stained with DAPI) and ROS (stained with DHE) also demonstrated that the ROS was mainly distributed in hepatocytes (Figure 5F) and bicyclol decreased the liver ROS accumulation triggered by poly(I:C)/D-GalN in a dose-dependent manner (Figure 5D, DHE staining and 5G). The ROS is distributed mainly in hepatocytes These results suggest that bicyclol ameliorates poly(I:C)/D-GalN-induced liver injury and inflammatory factor release, at least partly, by inhibiting ROS generation and the subsequent activation of the MAPK/NF-κB pathway. The conclusion from the hepatitis mice model agreed with that from the HCV-infected cells (Figure 3).

## Discussion

**Figure 6:**
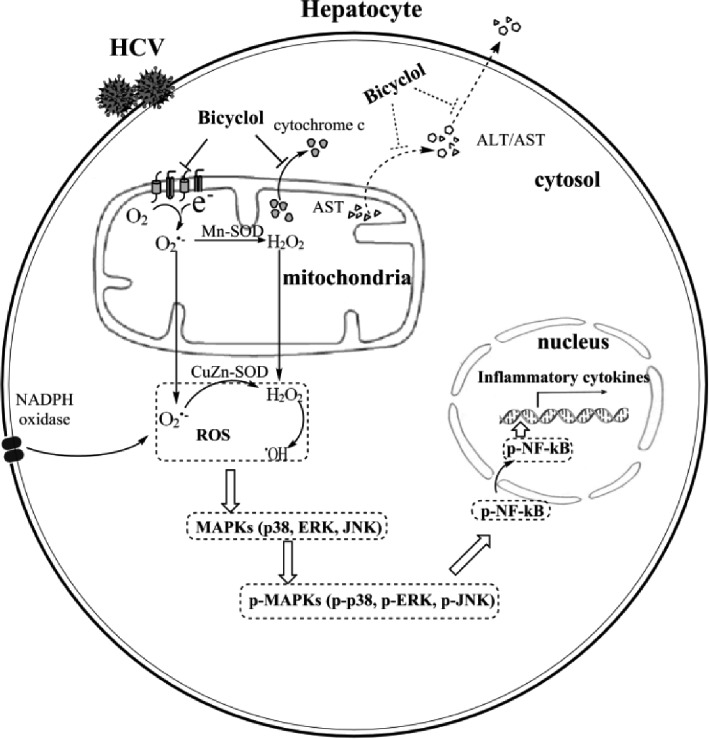
Schematic diagram of the anti-inflammatory mechanism of bicyclol. Bicyclol reverses the HCV-disturbed mitochondrial transmembrane potential, without altering the expressions and activities of NADPH oxidase and superoxide dismutases, decreases the cellular ROS levels, and inhibits the subsequent MAPK and NF-κB activation. The overall anti-inflammation and hepatoprotection effects of bicyclol are characterized by the decreased inflammatory cytokine production and the mitochondrial cytochrome c release to the cytosol and/or the ALT/AST release into the extracellular matrix. The dotted line shows the potential mechanism not verified in our study.

Bicyclol was widely used in China for the treatment of various types of liver injury, including hepatitis B and C (Guo et al., 2003; Liu, 2009). Its mechanism involves mitochondrial protection, antioxidant stress, anti-apoptosis, or/and toll-like receptor pathway inhibition (Liu, 2009; Zhang et al., 2016). However, the anti-inflammatory mechanism of bicyclol in hepatitis C remains to be clarified. Once infected with HCV, ~80% of individuals develop chronic hepatitis C naturally (Leone and Rizzetto, 2005). HCV triggers the occurrence of oxidative stress and the overproduction of inflammatory factors and, thus, facilitates disease progression and even leads to HCC (Nakamoto and Kaneko, 2003). In this study, we demonstrated that bicyclol significantly inhibited the inflammatory response in HCV-infected hepatocytes and improved liver injury in hepatitis mice, and the combined use of bicyclol with antivirals produced a synergistic anti-inflammatory effect. Detailed mechanism studies showed that bicyclol decreased the level of HCV-induced ROS by recovering the mitochondrial function independent of NADPH oxidase and superoxide dismutases and, thus, inhibited the HCV-induced inflammation by blocking the ROS-MAPK-NF-κB pathway (Figure 6). Moreover, after HCV clearance by treatment with DAAs, the increased level of inflammatory factors is still persistent (Figure 1D), suggesting that the restoration of liver injury is delayed following HCV elimination. This may be a reason that persistent disease progress is still persistent after finishing antiviral therapy in the clinic (Conti et al., 2016; Reig et al., 2016; Jacobson et al., 2017), and thus, the anti-inflammatory therapy regimen is still very important for those patients.

Clinical evidence suggests that the long-term application of bicyclol might, to some extent, decrease the HCV load (Guo et al., 2003; Liu, 2009). Our results demonstrated that bicyclol’s anti-inflammatory effect was distinguished from its anti-HCV roles (Figure 2), although its anti-HCV role might contribute to its anti-inflammatory effect. Transcription factors, such as NF-κB, AP-1, and STAT3, regulate the production of proinflammatory cytokines and chemokines during HCV infection, among which NF-κB plays a central role in the inflammatory response (Gong et al., 2001; Hassan et al., 2007). Therefore, we focused on the NF-κB-mediated inflammation response during HCV infection. Our data demonstrated that the inflammation inducer is HCV itself but not the inflammatory mediators in the culture supernatants (Figure 1C). A report showed that HCV infection induced the nuclear accumulation of NF-κB p65 and the subsequent activation of inflammatory response in TLR3-reconstituted hepatocytes (Huh7.5-TLR3 cells) but not in Huh7.5 cells (Li et al., 2012). However, Huh7.5 cells are a cell line with deficient TLR3 expression (Sumpter et al., 2005; Prescott et al., 2007), and we still detected the inflammatory factor storm and an increased level of p-NF-κB, with an unchanged level of total NF-κB (Figure 1A,B) during HCV infection, which was consistent with previous reports (Lin et al., 2010, 2011).

MAPK (p38, ERK, and JNK) phosphorylation is also widely reported to activate downstream of the IKK-IKB-NF-κB pathway (Lin et al., 2010; Das and Ghosh, 2017). NF-κB activation, induced by HCV, is accompanied by increased oxidative stress, which is characterized by enhanced intracellular ROS levels and the subsequent activation of the MAPK pathway (Koike and Miyoshi, 2006; Fujinaga et al., 2011). Additionally, the ROS-MAPK-NF-κB axis is also reported in other inflammatory models induced by LPS, poly(I:C), and virus (Liu et al., 2008; Lin et al., 2010; Hsu et al., 2013; Li et al., 2016). Our data from the HCV-infected Huh7.5 cells are consistent with these studies (Figure 3). Bicyclol inhibited the activation of the HCV-induced MAPK pathway, leading to decreased p-NF-κB levels (Figure 3B), and this effect might be through decreasing cellular superoxide levels (Figure 3A), which was confirmed in the NF-κB-Luc dual luciferase reporter system (Figure 3C). This might be the reason that bicyclol is efficacious in various hepatitis types because oxidative stress universally exists in patients.

Oxidative stress is generally induced during HCV infection, and the mitochondria are the main source of cellular superoxide (Brault et al., 2013). Our result showed that bicyclol decreased the HCV-induced ROS level by restoring the HCV-reduced mitochondrial transmembrane potential (Figure 4A), which further decreased abnormally the cytochrome c release into the cellular cytosol (Figure 4B). HCV-induced ROS generation was also reported to improve Nox1 and Nox4 expression in HCV-infected Huh7 cells or in HCV RNA-transfected HepG2 cells (Deng et al., 2008; Boudreau et al., 2009; de Mochel et al., 2010). However, in our infectious system, HCV infection only slightly increased the expression of Nox4 but not Nox1 (Figure 4C), while Nox activity was significantly increased after HCV infection (Figure 4D). This phenomenon might be due to the different hepatocytes and HCV infection systems, as HCV cDNA-transfected HepG2 cells displayed a basal level of Nox1 mRNA expression, and only a modest Nox4 mRNA level change (<10%) was detected in the JFH-AM2 HCV clone-infected Huh7 cells (Boudreau et al., 2009). Previous studies also suggest that Nox-dependent innate immune responses are less robust in the Huh7.5 cell line (Boudreau et al., 2009). In addition, Mn-SOD or CuZn-SOD, which degrades superoxide anion into H_2_O_2_, also affects cellular superoxide accumulation, and the effect on Mn-SOD or CuZn-SOD after HCV infection is controversial (Abdalla et al., 2005; Lee et al., 2016). Our result showed that HCV infection and bicyclol treatment had no effect on their expression and activities (Figure 4C,E). These data suggest that bicyclol decreases the level of HCV-induced ROS by restoring mitochondrial function without a dependence on NADPH oxidase and superoxide dismutases. However, the detailed mechanisms still need to be clarified.

Persistent intrahepatic inflammation and hepatocellular apoptosis lead to liver injury and even to HCC (Nakamoto and Kaneko, 2003). Poly(I:C), a synthetic analog of viral double-stranded RNA, is commonly used to study the RNA viral infection-induced immune response (Takahashi et al., 2006; Liu et al., 2008). It also induces a MAPK-dependent expression of proinflammatory cytokines and chemokines in cells (Liu et al., 2008). D-GalN is a hepatotoxic agent, which induces liver damage that closely resembles human viral hepatitis (Decker and Keppler, 1972). Previous reports suggest that D-GalN triggers liver ROS production, activates the MAPK signaling pathway in rats, and facilitates mitochondrial apoptosis to contribute to producing oxidative stress and inflammation in the liver (Quintero et al., 2002; Lin et al., 2009; Ganai et al., 2015). Coinjection of D-GalN and poly(I:C) synergistically mediates the severe liver injury (Dejager and Libert, 2008). Bicyclol significantly decreased poly(I:C)/D-GalN-induced liver injury and inflammatory response by reducing the ROS accumulation and the subsequent phosphorylation levels of p38, ERK, and NF-κB in this model (Figure 5), which was similar with the changes in the HCV-infected hepatocytes (Figure 3). Besides, the total NF-κB level in this mice hepatitis model also increased, which we speculated is caused by poly(I:C)/D-GalN induced damage-associated molecular patterns *in vivo*. The effect of bicyclol in our liver injury model is consistent with its hepatoprotection and anti-inflammation roles in other chemical and immunological liver injury mouse models (Liu, 2009). However, our data could not exclude the potentially direct role of bicyclol for blocking inflammatory factor triggered canonical NF-κB activation, and there needs more evidence for further confirmation.

## Conclusion

The present study reported that bicyclol attenuated the inflammation in HCV-infected hepatocytes and ameliorated liver injury in mouse hepatitis induced by the coinjection of poly(I:C) and D-GalN. The detailed mechanism showed that bicyclol decreased the level of HCV-induced ROS by recovering mitochondrial function without a dependence on NADPH oxidase and superoxide dismutases and, thus, inhibited HCV-induced excessive inflammation by blocking the activation of the ROS-MAPK-NF-κB pathway. Because this pathologic pathway is universal in a variety of hepatitis types and inflammation still exists in HCV-infected patients after they achieve an SVR, bicyclol might contribute to eventually eliminating hepatitis C.

## Author Contributions

HL designed and performed the experiments, analyzed the data and wrote the manuscript. J-RL and M-HH performed the experiments and analyzed the data. J-HC, X-QL, L-LZ, J-LT, and BD performed the experiments. J-DJ and Z-GP oversaw the project, designed the experiments, analyzed the data, and wrote the manuscript.

### Conflict of Interest Statement

The authors declare that the research was conducted in the absence of any commercial or financial relationships that could be construed as a potential conflict of interest.
